# Disaster and Distress: The Double Burden. Depression, Anxiety, and Post-traumatic Stress Disorder in Healthcare Providers during the COVID-19 Pandemic in Pakistan

**DOI:** 10.1192/j.eurpsy.2025.1269

**Published:** 2025-08-26

**Authors:** M. Fatima, N. Imran, I. Aamer, S. Iqtadar, B. Shabbir

**Affiliations:** 1 Academic Department of Psychiatry and Behavioral Sciences, King Edward Medical University Lahore, Lahore, Pakistan; 2 Psychiatry, Dublin North Mental Health Service, Dublin, Ireland; 3 Child and Family Psychiatry Department; 4 General Medicine, King Edward Medical University Lahore; 5 General Medicine, Fatima Jinnah Medical University, Lahore, Pakistan

## Abstract

**Introduction:**

Healthcare providers are at a high risk of occupational stress, psychological distress, and mental health problems due to unique job demands. The unprecedented negative impact of the COVID-19 pandemic on healthcare further intensified the risk.

**Objectives:**

To find out the prevalence of anxiety, depression, and post-traumatic stress disorder (PTSD) in healthcare providers during the COVID-19 pandemic, and explore various associated factors.

**Methods:**

We conducted a cross-sectional survey among clinicians across tertiary care hospitals in Lahore, Pakistan through online forms/ paper-based questionnaires, using the Patient Health Questionnaire (PHQ-9), Generalized Anxiety Disorder (GAD-7) Scale, and The Impact of Events Scale-Revised (IES-R) from July-November 2021. Physicians aged 20-60 years working at the public-sector hospitals of Lahore were included through non-probability convenient sampling. The non-parametric Mann-Whitney *U, Kruskal-Wallis, and Chi-square* test of independence were applied for inferential data analysis in SPSS version 26 at α=0.05.

**Results:**

Of 302 participants, the majority were males (54.6%), from medicine and allied departments (76.9%), junior staff (72%), and frontline workers (75%). 9.5% had a history of psychiatric illness. The prevalence of depression was 25%, anxiety 31.9%, PTSD 15.8%, severe depression 13.8%, and severe anxiety 7.2%. The total median (IQR) scores of depression, anxiety, and PTSD were 5(2-9.5), 4(0-7), and 10(1-23) respectively. Females and junior staff had comparatively severe symptoms of anxiety and depression. Psychiatric history was linked to severe depression (p=0.003) and PTSD (p=0.001) but not anxiety (p=0.136). There were no statistically significant differences in the anxiety and PTSD severity across genders, departments, and frontline/second-line work.

**Conclusions:**

We found high levels of depression, anxiety, and PTSD in our physician sample even during the 4^th^ COVID-19 wave. This has implications for emphasizing the significance of the mental well-being of healthcare providers and identifying effective interventions to prioritize it even after the pandemic.

**Disclosure of Interest:**

None Declared

